# Asymmetric Divergence in Structure and Function of HCN Channel Duplicates in *Ciona intestinalis*

**DOI:** 10.1371/journal.pone.0047590

**Published:** 2012-11-02

**Authors:** Heather A. Jackson, Andrew Hegle, Hamed Nazzari, Timothy Jegla, Eric A. Accili

**Affiliations:** 1 Department of Cellular and Physiological Sciences, University of British Columbia, Vancouver, British Columbia, Canada; 2 Department of Biology, Eberly College of Science, Pennsylvania State University, University Park, Pennsylvania, United States of America; Virginia Commonwealth University, United States of America

## Abstract

Hyperpolarization-activated Cyclic Nucleotide (HCN) channels are voltage-gated cation channels and are critical for regulation of membrane potential in electrically active cells. To understand the evolution of these channels at the molecular level, we cloned and examined two of three HCN homologs of the urochordate *Ciona intestinalis* (*ciHCNa* and *ciHCNb*). ciHCNa is like mammalian HCNs in that it possesses similar electrical function and undergoes N-glycosylation of a sequon near the pore. ciHCNb lacks the pore-associated N-glycosylation sequon and is predictably not N-glycosylated, and it also has an unusual gating phenotype in which the channel's voltage-sensitive gate appears to close incompletely. Together with previous findings, the data support an evolutionary trajectory in which an HCN ancestor underwent lineage-specific duplication in *Ciona*, to yield one HCN with most features that are conserved with the mammalian HCNs and another HCN that has been uniquely altered.

## Introduction

HCN channels are voltage-gated cation channels with an unusual set of functional properties that make them critical for regulating membrane potential in electrically active cells such as neurons and cardiomyocytes [1]. Principally, HCNs are opened by hyperpolarization of the membrane potential, allow both sodium and potassium ions to pass and most isoforms open more easily when cAMP is bound to their cytoplasmic surface. To date, HCNs have been cloned from mammals (HCN1-HCN4) [2], [3], [4], arthropods (one gene) [5], [6], [7], [8] and sea urchin (two genes) [9], [10]. All known HCN genes predict channels comprised of a voltage sensing domain, an ion conducting pore region and a cyclic nucleotide binding domain in the carboxyl-terminus.

Functionally, HCNs cloned from mammals and invertebrates differ quantitatively [11]. Among the four mammalian HCNs, opening occurs at different rates and, notably, is facilitated strongly by cAMP in only two isoforms (HCN2 and HCN4) [12], [13], [14], [15], [16], [17]. The one sea urchin isoform whose function is known opens and closes comparably quickly [9] but its internal gate re-closes during hyperpolarization-induced opening [18]; reclosing no longer occurs when cAMP is bound. Other invertebrate HCNs are more vertebrate-like; they do not re-close and open more easily when cAMP is elevated [6], [7], [8]. Finally, the vertebrate, but not invertebrate, HCNs also contain a putative N-linked glycosylation site near the pore [19], which has been shown to control cell surface expression variably among the four mammalian isoforms [20], [21].

*Ciona intestinalis* is a marine organism whose tadpole-like larval stage undergoes a phase of metamorphosis and gives way to a sessile adult stage adapted for filter feeding [22]. It is a deuterostome and is classified as a marine invertebrate chordate, or urochordate, [23], due to the presence of a notochord at the tadpole stage. *C. intestinalis* displays a simple adult body plan, which includes a heart with an open circulatory system, a neural complex, and digestive and reproductive organs. This organism diverged from the vertebrate lineage over 550MYA and is part of the chordate phylum along with other urochordates (ascidians, larvaceans and thaliaceans), cephalochordates (amphioxus) and vertebrates [24]. Thus, urochordates provide a close ancestral genetic reference point prior to the duplication and diversification events that have occurred in vertebrate genomes [25].

A comprehensive analysis of the *C. intestinalis* genome revealed 160 ion channel genes with homologs in mammals [26], which include a minimal set of voltage-gated ion channel genes. Among these voltage-gated channels are three hyperpolarization-activated cyclic-nucleotide modulated (HCN) channels [26]. We identified and analyzed the HCN homolog sequences in two tunicate genomes, *Ciona intestinalis* and *Ciona savignyi* [19], which are very similar to each other in sequence and phylogenetically group together as an independent clade, rather than as orthologs of the four vertebrate isoforms. This pattern suggests that the three *Ciona* HCN genes arose via lineage-specific duplications prior to divergence of *C. intestinalis* and *C. savignyi*. Based on exon structure, sequence identity and phylogenetic position, we suggested that ciHCNb is evolutionarily closest to the common ancestor, while ciHCNa and ciHCNc arose through subsequent lineage-specific duplications [19]. ciHCNa and ciHCNb both share similar sequence identity with invertebrate HCNs (approximately 55%) and vertebrate HCNs (approximately 75%). ciHCNc shares less sequence similarity with these two groups, approximately 40% and 60%, with invertebrate and vertebrate HCNs, respectively.

We suspected that the function of ciHCNa and ciHCNb would be more similar to the vertebrate HCNs than ciHCNc because the amino acid sequence of the narrowest part of the pore responsible for ion selectivity and flow is the same between the vertebrate and former *Ciona* HCNs. But, between ciHCNa and ciHCNb, the degree of functional similarity to the vertebrate HCNs is difficult to predict. Although ciHCNb possesses an exon structure that is mildly more similar to the vertebrate HCNs [19], ciHCNa shares the pore-associated N-glycosylation sequon with vertebrate HCNs. Here, we have cloned and explored the function of ciHCNa and ciHCNb. Our analysis suggests that a lineage-specific duplication in *Ciona* yielded one HCN with most features conserved with those of the mammalian HCNs (ciHCNa) and another HCN that has been uniquely altered (ciHCNb).

## Materials and Methods

### HCN sequence collection and analysis

**Figure 1:**
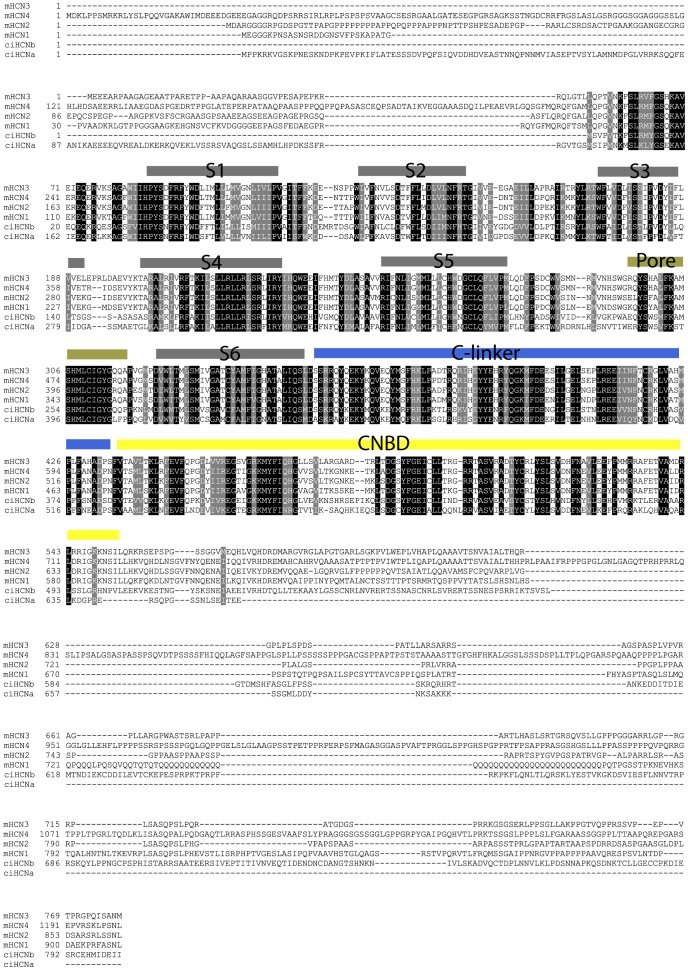
Ciona HCNs show conservation of key regions, but also considerable divergence, with each other and with the mammalian HCN isoforms. Shown is an alignment of ciHCNa and ciHCNb with the four HCN isoforms from the mouse. Identical (black) and conserved (gray) residues among all of the isoforms are shaded. The approximate locations for the six putative transmembrane segments, the pore, C-linker and cyclic nucleotide-binding domain are identified and indicated using solid bars place above the sequence. The sequences shown are: ciHCNa (GI:378407787), ciHCNb (GI:378407789), mouse HCN1 (GI:255760033), mouse HCN2 (GI:6680189), mouse HCN3 (GI:6680191) and mouse HCN4 (GI:124487125). The alignment was carried out using Clustalw (http://bio.lundberg.gu.se/edu/msf2.html) and arranged into the figure by Boxshade 3.21 (http://www.ch.embnet.org/software/BOX_form.html).

**Figure 2:**
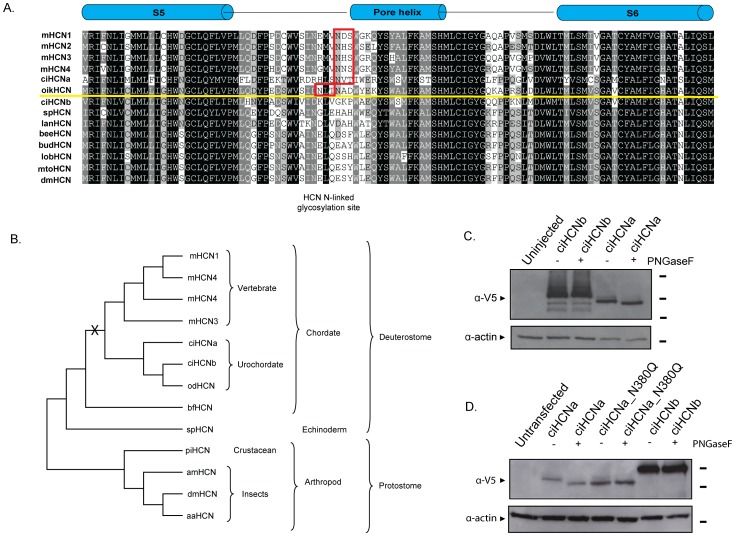
Phylogenetic pattern and N-linked glycosylation of a subset of *Ciona* HCNs. A. An alignment of the pore region produced by ClustalX and generated by GeneDoc. Red boxes indicates known and putative HCN N-linked glycosylation site and the yellow line indicates the division between the presence and absence of this functional site. B. An HCN cladogram generated by aligning the sequences shown as described in the text. “X” indicates the predicted emergence point of N-linked glycosylation in the HCN family. Sequences other than those in Figure 1, and other than those for *Oikopleura dioica* and *Branchiostoma floridae* (lancelet), are: gi: 260829977; *Strongylocentrotus purpuratus* (sea urchin) gi:47551101; *Panulirus interruptus*, splice variant I (lobster) gi:115334851; *Apis mellifera* (bee) gi:33355927; *Drosophila melanogaster*, isoform B, gi:84795752; *Aedes aegypti* (mosquito) gi:108875949. C. Western blot of membrane fractions from oocytes injected with cRNA of ciHCNa, ciHCNa-N380Q or ciHCNb, untreated (−) or treated (+) with PNGaseF and probed with anti-V5 antibody. Actin, robed with an anti-actin antibody, was used as a loading control. ciHCNa, but not ciHCNa-N380Q or ciHCNb, is shifted to a lower molecular mass in the presence of PNGaseF. Molecular weights, indicated by the short black bars to the right of the blots, are 130 kDa (top), 100 kDa (upper middle) and 70 kDa (lower middle) and 35 kDa (bottom). D. Western blot of whole cell lysates from CHO cells transfected with 1.5 ug cDNA of ciHCNa, ciHCNa-N380Q or ciHCNb, either treated (+) or untreated (−) with PNGaseF and probed for using an anti-V5 antibody. Molecular weights, indicated by the short black bars to the right of the blots, are 95 kDa (top), 72 kDa (middle) and 34 kDa (bottom). Predicted mass of *Ciona* HCN channels without post-translational modification, is 78.8 kDa and 93.3 kDa for ciHCNa and ciHCNb, respectively.

For *Branchiostoma floridae* and *Oikopleura dioica*, HCN sequences were obtained by a BLAST/BLAT search of their respective genomic databases (*Oikopleura dioica* v1 at www.genoscope.cns.fr) [27]. A segment of the genomes near the search hits was downloaded and run through a genewise prediction program. Other sequences were obtained from NCBI and their accession numbers are included in legends for Figures 1,2. Translated protein sequences were aligned with ClustalX [28]; the NH2 and COOH- terminal regions were subsequently removed, leaving a region between the S1 and end of the CNBD. The phylogenetic tree was determined using a neighbor joining tree method and MEGA 5.0 software [29]; the approach is a simplified version of the minimum evolution method and the tree produced is unrooted [30].

### Cloning and epitope tagging of two Ciona HCN channels

We initially cloned and chose to analyze ciHCNa and ciHCNb because, unlike ciHCNc, both retain pore sequences that are highly conserved among all known HCNs [19]. However, only ciHCNa possesses a putative N-glycosylation sequon near the pore, as do most other vertebrate sequences. Sequences were identified by BLAST search of the *Ciona intestinalis* genome sequence [31] using mouse HCN1 as a query. The ciHCNa coding sequence was determined by comparison of gene predictions and sequenced cDNA clones, and cloned by standard RT-PCR from *Ciona intestinalis* total RNA isolated from adult siphon. Briefly, 5 µg of total RNA was reverse transcribed using Superscript III (Invitrogen, Carlsbad, CA) and oligo-dT priming. 1/50^th^ of this reaction was used for each RT-PCR. The gene was amplified in two pieces, assembled by standard overlap PCR and cloned into pOX for expression in oocytes [32], [33], or pCDNA3.1 derivative, which expresses eGFP off the same transcript under control of the CMV promoter for expression in mammalian cells. The 5′ end of ciHCNb could not be determined from gene predictions or cDNA clones and was identified by 5′ RACE PCR using the Marathon cDNA synthesis kit (Clontech, Palo Alto, CA) with total siphon RNA as a template. Full length ciHCNb clones for expression were then assembled from three pieces by overlap PCR and cloned as described above for ciHCNa. 4 separate clones were sequence verified for ciHCNa and ciHCNb; only clones that fully matched the consensus amino acid sequence determined for each gene were used for functional experiments.

A C-terminal V5-tagged ciHCNa and ciHCNb was constructed by overlapping PCR mutagenesis and digested back into the original pOX vector for oocyte expression or into a pcDNA3.1 myc (Invitrogen) vector using BamHI and Not1 restriction enzymes. The N380Q ciHCNa-V5 mutant was then constructed using QuickChange mutagenesis. Resulting sequences were confirmed by an automated sequencing facility (UBC).

### Identification of N-glycosylation of Ciona HCN channels in Xenopus oocytes and Chinese Hamster ovary cells

To identify N-glycosylated protein, channels were expressed in *Xenopus* oocytes or Chinese Hamster ovary cells. Oocytes were obtained in the following way. After housing *Xenopus laevis* frogs in a habitat-enriched environment to allow normal behaviors and enhance stimulation, anesthesia was induced by immersing in them in 1 g/litre tricain methanesulphonate anaesthetic solution (buffered to neutral pH with HEPES) for 30–45 minutes. Anaesthesia was confirmed by the absence of toe pinch reflex, the corneal reflex and righting reflex. Euthanasia was carried out by severing the spinal cord and pithing the brain. Following a midline incision, the lobes of oocytes were dissected from the body cavity and separated into small clumps of 5–10 oocytes. All procedures involving live animals conformed to guidelines established by the Canadian Council for Animal Care and the University of British Columbia Committee on Animal Care, and were approved by the latter.

For oocyte expression, plasmids were linearized with Not1 and mRNA was produced by in vitro transcription from the T3 promoter (Message Machine kit, Ambion). Oocytes were washed with OR2 solution (82.5 mM NaCl, 2.5 mM KCl, 1 mM MgCl2 and 5 mM HEPES, pH 7.6 with NaOH) and dissociated using 2 mg/ml collagenase 1A (Sigma Aldrich) in OR2 for at 1 hr. Oocytes were then washed 3× with OR2 solution, followed by 3× with OR3 solution and incubated in OR3 solution for at least 1 hr. Mature decollagenated oocytes were injected with 25 ng of the indicated RNAs and maintained in OR3 media at 18°C. 48 hrs post-injection, 40–50 oocytes for each condition were homogenized on ice in HEDP buffer (100 mM HEPES, 1 mM EDTA, pH 7.6) with protease inhibitors (10 µg/mL each of aprotinin, pepstatin, and leupeptin and 1 Roche protease inhibitor tablet per 10 mL) and centrifuged 2× at 6,000 RPM for 2 min at 4C. Supernatants were then overlaid on a 15% sucrose/HEDP cushion and ultracentrifuged at 50,000 RPM for 90 min at 4C to isolate membrane fractions. Pellets were resuspended in HEDP buffer and protein concentrations were determined by Bradford assay.

Chinese hamster ovary-K1 (CHO) cells (American Tissue Type Culture Collection) were maintained at a sub-confluent density in F-12 media supplemented with 10% FBS and were transiently transfected with indicated cDNAs with FuGENE, according to manufacturer protocols (Roche). After 24 hrs, cells were washed with PBS and lysed for 30 min in RIPA buffer containing, in mM: 50 Tris (pH 8.0), 150 NaCl, 1 EDTA, 1 PMSF, 2 Na_3 VO_4, 2 NaF, 1% NP40 and 10 µg/mL each of aprotinin, pepstatin, and leupeptin. Protein concentration was determined by Bradford assay (BioRad). Proteins were resolved using SDS-PAGE and transferred to polyvinylidene difluoride (PVDF) membranes. Blots were probed with antibodies for V5 (1∶2000) and actin (1∶5000), followed by HRP-conjugated secondary antibody (1∶2000), and visualized with ECL (Amersham). For PNGaseF assays (New England Biolabs), extracts were first incubated with PNGaseF for 1 hr at 37°C before SDS-PAGE.

### Electrophysiological analysis of Ciona HCNs in Xenopus oocytes

Oocytes were injected with 50 nl of mRNA (5–50 ng total) and stored at 18°C in OR3. In oocytes expressing either *Ciona* HCN isoform, ionic current was recorded with the two-microelectrode voltage clamp technique using an OC-725C voltage clamp (Warner Instruments, Hamden, CT), 1–3 days after injection. Borosilicated glass microelectrodes (Sutter Instruments Co.) were filled with 3 M KCl and had a resistance of 0.5 to 1.5 Mohms. Liquid junction potential was compensated prior to oocyte entry. Data was acquired using an amplifier and 1440 analagoue/digital converter, along with pClamp 10.0 software. All experiments were carried out at room temperature. Analysis was done using Clampfit 10 (Molecular Devices), Origin (Microcal) and GraphPad software.

## Results

### Cloning and sequence analysis of two HCN channels from Ciona intestinalis

To directly examine them, two urochordate cDNA homologs of the mammalian hyperpolarization-activated cyclic nucleotide modulated (HCN) channel family were cloned from the adult siphon of *Ciona intestinalis* using genomic comparisons and PCR strategies. We have termed these clones ciHCNa and ciHCNb to adequately differentiate them from the HCN1-4 mammalian isoforms and they have been deposited in GenBank.

An alignment of the region between the beginning of the first transmembrane segment and the end of the cyclic nucleotide binding of ciHCNa and ciHCNb with four mammalian isoforms is shown in Figure 1. Key domains are well-conserved between the *Ciona* and mouse isoforms including the voltage-sensing region (S4 segment), the pore, the region containing the putative internal gate (S6), the cyclic nucleotide-binding domain (CNBD) and the C-linker, which connects the pore and internal gate of the pore to the CNBD. Within the entire sequence shown, the *Ciona* HCNs share approximately 50% sequence identity with the mammalian isoforms. ciHCNa is 671aa, 17 amino acids shorter than the predicted gene (JGIv1 ID: ci0100152577), due to a deletion in the distal C-terminus of the channel. Thus, the version of ciHCNa we cloned may represent a splice variant. Otherwise, the cloned sequence is 97% identical to that predicted in the genome. ciHCNb is 802 aa long, also 97% identical to the genome predicted sequence (JGIv1 ID: ci0100130432)

Within the first transmembrane segment and the end of the CNBD, ciHCNa and ciHCNb are only approximately 55% identical to each other, and show even less conservation within this conserved region to the putative ciHCNc gene. This contrasts with the mammalian HCN isoforms, which share between 80 and 90% of coding sequence among the four genes [19]. The comparatively larger differences among the *Ciona* HCN isoforms suggests that evolution at the molecular level has occurred more quickly or over a longer period of time. When compared to *C. savignyi*, ciHCNa and ciHCNb are 90% and 93% identical within the region comprising the first transmembrane segment and the end of the CNBD.

### Phylogenetic tracking of the pore-linked N-linked glycosylation sequon

Previously, we were struck by our finding that a N-linked glycosylation site (NXS/T), in the extracellular region near the pore, is found in HCNs of vertebrates and two of three *Ciona* HCNs (ciHCNa and ciHCNc), but not in cnidarians, arthropods, annelids or mollusks [19], [20]. Here, we examine the phylogenetic pattern of HCN evolution among chordates by aligning the region of containing the N-glycosylation sequon (Figure 2A) and generating an unrooted cladogram (Figure 2B) of a sub-set of HCNs that includes sequences from the tunicate *Oikopleura dioica* and from *Branchiostoma floridae* (also known as amphioxus or lancelet). Other than the *Oikopleura* and *Branchiostoma* HCNs, the cladogram expectedly recapitulates the phylogeny described in our previous study, which was determined by several methods and utilized large number of sequences [19]. The cladogram also shows that the sole HCN channel from *Branchiostoma floridae* falls between that of urochordates and echinoderms, while an alignment of key sequences shows it does not have the pore-associated N-glycosylation sequon found in other vertebrate and *Ciona* HCNs. The sole HCN from *Oikopleura dioica* groups with the *Ciona* HCNs, most closely to ciHCNb, and possesses an N-glycosylation sequon near the pore, just N-terminally adjacent to the conserved location in the alignment.

These findings provide support for a pattern in which the N-glycosylation sequon arose in a common ancestor of urochordates and vertebrates, but was lost in ciHCNb. The more distant position of *Branchiostoma* HCN within the tree, and its lack of the N-glycosylation sequon, lends some support to a recent evidence suggesting that tunicates, and not amphioxus, are the closest relatives of vertebrates [24], [34]. Although the N-glycosylation sequon of ciHCNa is found in the same location as in the vertebrate HCNs, and also appears near the pore of both ciHCNc and the sole HCN from *Oikopleura dioica*, it is possible, but less parsimoniously likely, that it appeared twice independently; once in an ancestor common to all tunicates and another time in an ancestor common to all vertebrates.

### N-glycosylation of ciHCNa, but not ciHCNb, when expressed in cells

To confirm whether either ciHCNa or ciHCNb can be N-glycoslyated, they were tagged with a C-terminal V5 epitope and expressed in both oocytes and mammalian cells. Membrane fractions were either left untreated or treated with PNGaseF and run on an SDS-PAGE gel. Indeed, in both CHO cells and *Xenopus* oocytes (Figure 2C and 2D, respectively), ciHCNb is present mainly as one PNGaseF-insensitive band, suggesting that this isoform, which lacks the pore-associated N-glycosylation sequon, is predictably not N-glycosylated. In contrast, ciHCNa is predominantly one band on the gel that is shifted almost completely to a lower molecular weight when PNGase is added, suggesting that this channel exists primarily in its N-glycosylated form in both cell types. Upon mutation of the putative N-glycosylated asparagine to glutamine, the resulting ciHCNa band was shifted to the same weight as that for the wild type channel after PNGase treatment, and its position was unaltered by exposure to that agent. This is good evidence that the pore associated sequon in ciHCNa is the sole N-glycosylated site within this channel.

Interestingly, the molecular weights for the *Ciona* HCNs appear to be slightly larger when expressed in oocytes. The overall larger molecular weights of the *Ciona* HCNs extracted from oocytes may be due to a true difference because of, for example, a post-translational modification that occurs only in the oocyte or to a difference in the ability of the oocyte-extracted *Ciona* HCNs proteins to migrate. Nevertheless, the difference in molecular weight between ciHCNa and ciHCNb is approximately 15 kDa for both blots, consistent with the difference between their predicted molecular weights.

### Two Ciona HCNs form channels which are variably opened by hyperpolarization and blocked by cesium

**Figure 3:**
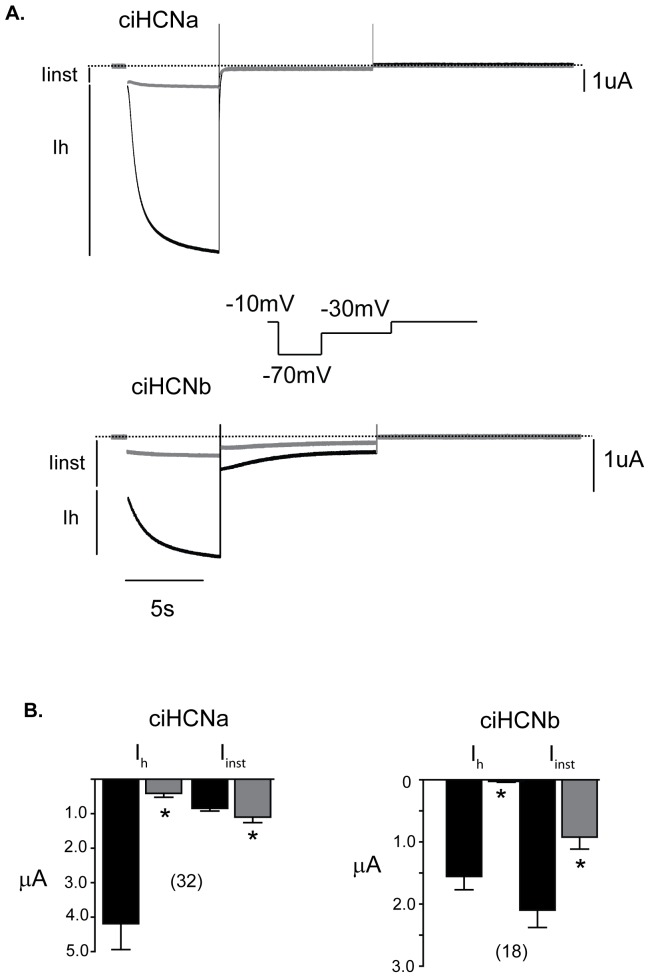
Variable opening by hyperpolarization and cesium block of current produced by the two *Ciona* HCNs. A. Black current traces elicited by a single 6 second hyperpolarizing pulse to −70 mV, from a holding potential of −10 mV, followed by pulse back to −30 mV, in an oocyte containing ciHCNa (above) or ciHCNb (below), bathed an extracellular solution containing 96 mM potassium. The voltage protocol utilized is shown between the two sets of current traces. The black vertical lines to the left indicate the instantaneous (I_inst_) and the slowly-activating (I_h_) components of the current trace. Gray current traces were produced by the same voltage protocol, following incubation with the same extracellular solution, with the addition of 5 mM Cs+, for three minutes while clamped at −10 mV. The dotted lines represent the zero current level. Capacitive transients elicited upon current activation have been removed for clarity. B. Plots of amplitudes of I_inst_ and I_h_ before and during perfusion with Cs+, as indicated. The number of oocytes that were used in each group are shown above each bar in brackets. Values represent mean ± s.e.m.; those in black and gray represent with and without Cs+. *indicates a significant difference induced by Cs+ (p<0.05 in a two-tailed paired t-test).

Both ciHCNa and ciHCNb are phylogenetically equidistant from, and share approximately the same sequence identity with, vertebrate HCNs [19]. However, ciHCNa is N-glycosylated, as are the vertebrate HCNs; this suggests it may have undergone less dramatic changes since duplication and, thus, it may be phenotypically closer vertebrate HCNs and their common ancestor. To compare their function with vertebrate HCNs, the electrical properties of the two *Ciona* HCN clones were established using two-electrode voltage clamp on *Xenopus* oocytes expressing either channel. In response to hyperpolarization of the membrane potential, both ciHCNs produced currents that are typical for all known HCN channels; namely a very fast or “instantaneous” current (I_inst_) followed by a slowly-activating current (I_h_ or I_f_) (Figure 3) [35], [36].

ciHCNa produces a relatively small I_inst_ and large I_h_, as is the case for most of the known HCNs. In contrast, ciHCNb produces an I_inst_ that is as large as I_h_. Application of extracellular cesium (Cs^+^), a known HCN channel blocker [37], eliminates I_h_ almost completely for both HCN isoforms. I_inst_ of ciHCNa is not greatly affected by Cs^+^, consistent with our findings in mammalian HCNs [35], [38]. The dramatic block of both the large I_inst_ of ciHCNb and I_h_ by Cs+ supports flow of the former through the pore itself, perhaps as a consequence of inadequate closing of the channel gate.

The amplitude of total hyperpolarization-activated current was not greatly different between the two *Ciona* HCNs even though there appeared to be larger amounts ciHCNb in the western blots in Figure 2. In our experience with overexpressed HCN channels from various species, we frequently observe that much of the protein does not reside at the plasma membrane and therefore there can be discrepancies between total protein level and current size. Assuming that the single channel conductance is similar between the two *Ciona* HCNs, we would suggest that more of ciHCNb resides within intracellular compartments.

### Permeation of Ciona HCNs is similar to known vertebrate and invertebrate HCNs

**Figure 4:**
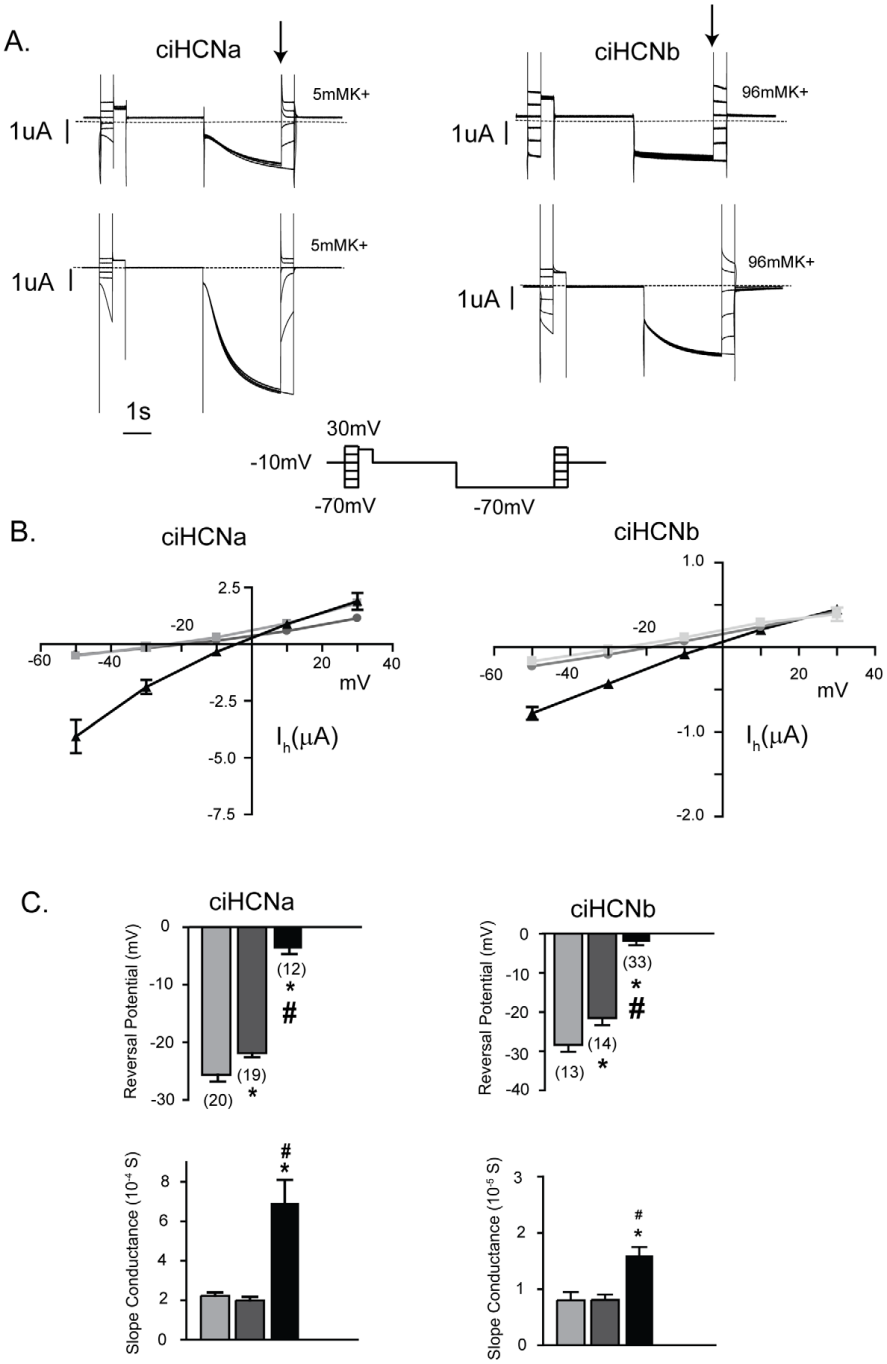
Potassium passes through both *Ciona* HCNs and enhances current flowing through them. A. Current traces elicited by a multi-step protocol in oocytes expressing either *Ciona* HCN isoform to obtain instantaneous I_h_ versus voltage relationship, carried out in low and high concentrations of extracellular potassium. The voltage protocol is shown below the current traces. The first set of voltage pulses allow for the determination of instantaneous current at each test voltage; the second set of pulses allows for the determination of total instantaneous current at each test voltage, following I_h_ activation at a potential at which the current is close to fully activated. Subtracting the latter from the former yields the total instantaneous I_h_ at each test voltage. B. Plots of instantaneous I_h_ amplitude versus test voltage at low and high concentrations of extracellular potassium as determined in ‘A’. Values represent mean ± s.e.m. at each test voltage; light gray/squares for low 5 mM K^+^, dark gray/circles for 10 mM K^+^, and (black/triangles) for 96 mM K^+^. C. Bar graphs showing the reversal potentials and slope conductance for each *ciona* HCN in different concentrations of potassium; these were determined from experiments in ‘A’ where values from individual experiments were fit by a straight line through the linear portion of the curve across the x-axis. The number of oocytes that were used in each group is shown above each bar in brackets; reversal potential and slope conductance were obtained from the same current data. Values represent mean ± s.e.m. for each potassium concentration. Statistical significance was determined using a one-way ANOVA and post-tukey multiple comparison test. * indicates difference between 96 mM potassium (p<0.05) and 5 mM and # indicates a difference between 96 mM potassium and 10 mM potassium (p<0.05). Light gray, dark gray and black bars represent values for 5, 10 and 96 mM potassium.

Known vertebrate and invertebrate HCN channels allow both potassium and sodium to pass; furthermore, they are more permeable to potassium and sodium requires potassium to pass [2], [3], [4], [6], [7], [9], [39], [40]. In general, raising extracellular K^+^, unlike Na^+^, also enhances current flow [37]. To examine the flow of sodium and potassium in ciHCNa and ciHCNb, we determined current-voltage relationships for I_h_, from which contaminating ion flow is easily separated. The I_h_ amplitude was determined by subtracting I_inst_ from the total current activated by hyperpolarization at each test voltage (Figure 4A). The I_h_ amplitude was then plotted against test voltage to yield current voltage relationships, from which the slope conductance and reversal potential was determined. For both *Ciona* HCNs, the current-voltage relationships was steeper and crossed the x-axis at less negative voltages in the high potassium-containing solution (Figure 4B,C), as has been described for other HCN-mediated currents [37].

**Figure 5:**
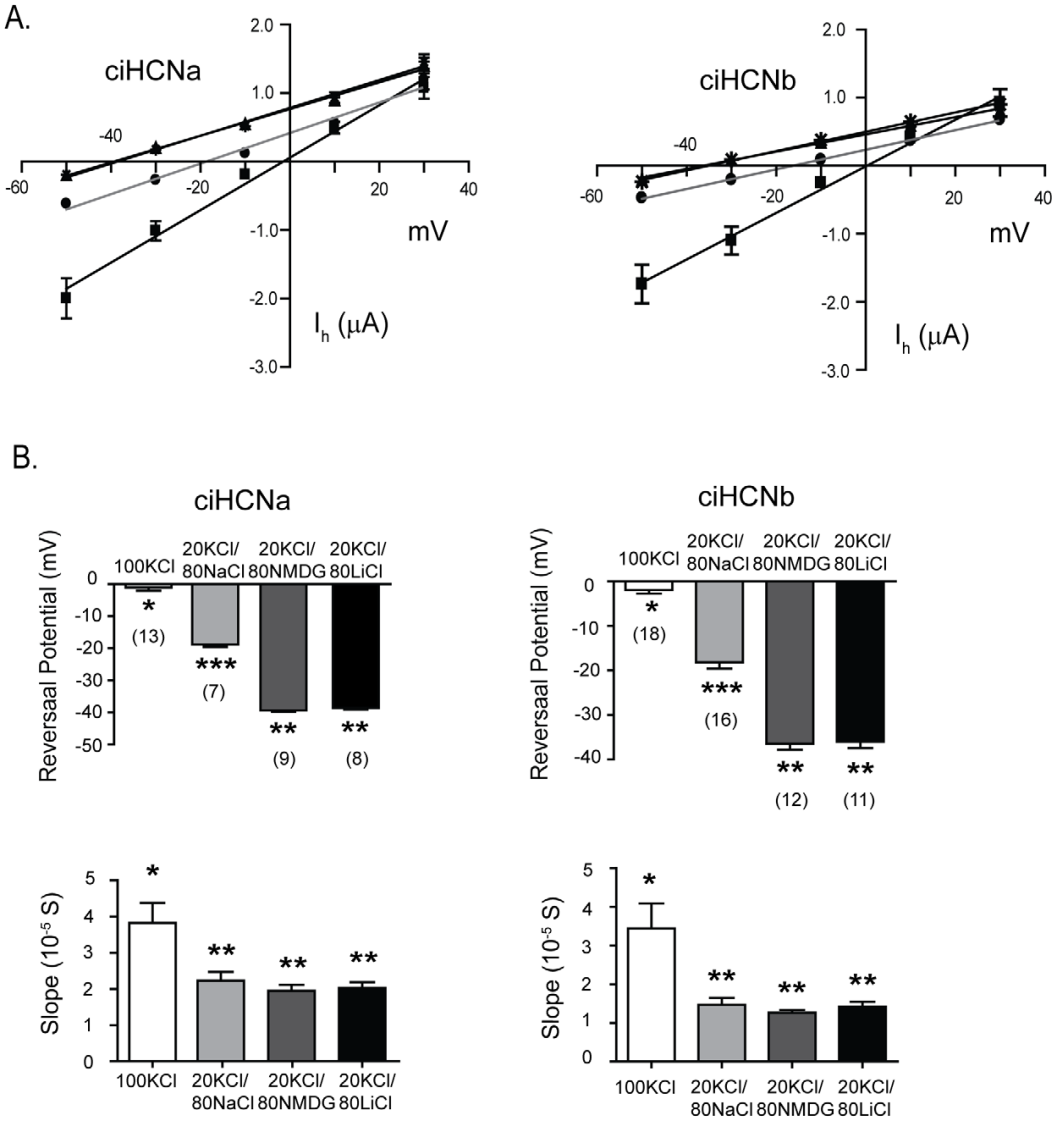
Sodium passes through both *Ciona* HCNs but does not enhance current flowing through them. A. Plots of instantaneous I_h_ versus voltage at the indicated concentrations of extracellular cations. Values were determined from current traces elicited using the same protocol as for Fig. 5 and represent mean ± s.e.m. for each test voltage; black line/black squares for 100 mM K^+^, light gray line/black circles for 80 mM Na+/20 mM K+, black line/black upward triangles for 80 mM Li/20 mM K+, black line/black downward triangles for 20 mM K+. Lines represent linear fits through the mean values. B. Bar graphs showing the reversal potentials and slope conductance for each *Ciona* HCN, determined from the plots in ‘B’ where values from individual experiments were fit by a straight line through the linear portion of the curve across the x-axis. The number of oocytes that were used in each group is shown below each bar in brackets; reversal potential and slope conductance were obtained from the same current voltage data. Values represent mean ± s.e.m. for solution used, which were compared using a one-way ANOVA and tukey's post test. * indicates a difference (p<0.05) when compared to all other conditions. ** indicates a difference (p<0.05) when compared to * or ***. *** indicates a difference (p<0.05) when compared to ** or *.

Because extracellular potassium enhances current amplitude, the extracellular solutions used to determine the relative permeability of sodium contained a constant level of KCl (20 mM) supplemented with 80 mM of one of KCl, NaCl, NMDGCl or LiCl (Figure 5). The current voltage relationship was approximately linear for all solutions, and was steeper and crossed the x-axis at less negative voltages in the high potassium-containing solution as compared to the low potassium containing solution. In extracellular solutions containing sodium chloride, the relationship, for both *Ciona* HCNs, crossed the x-axis at potentials more negative than when they contained 100 mM KCl, although not as negative as when the solution contained the impermeant NMDG. The shift of the reversal potential supports the passage of sodium through both ciHCNa and ciHCNb. A precise permeability ratio was not determined because the intracellular concentrations of sodium and potassium in the oocyte are not known when the two microelectrode voltage-clamp is utilized. In addition, the slopes of the relation are not greatly affected by sodium, unlike potassium, when compared to NMDG-containing solutions. The reversal potential in a solution containing lithium was more negative than those in solutions containing low potassium or sodium, and was the same as that for a solution containing NMDG. Thus, under our conditions, the permeation of lithium is minimal for both *Ciona* HCNs.

### For both Ciona HCNs, the range of I_h_ activation is depolarized by raising intracellular cAMP

HCN channels possess a domain in the C-terminus to which the intracellular messenger cAMP binds [2], [3], [9], [41], [42]. In some HCNs, binding of facilitates slow opening, which is manifested by a depolarizing shift in the range over which I_h_ activates, by relieving a tonic inhibition by the C-terminus [43], [44]; notably, cAMP itself does not open the channels.

**Figure 6:**
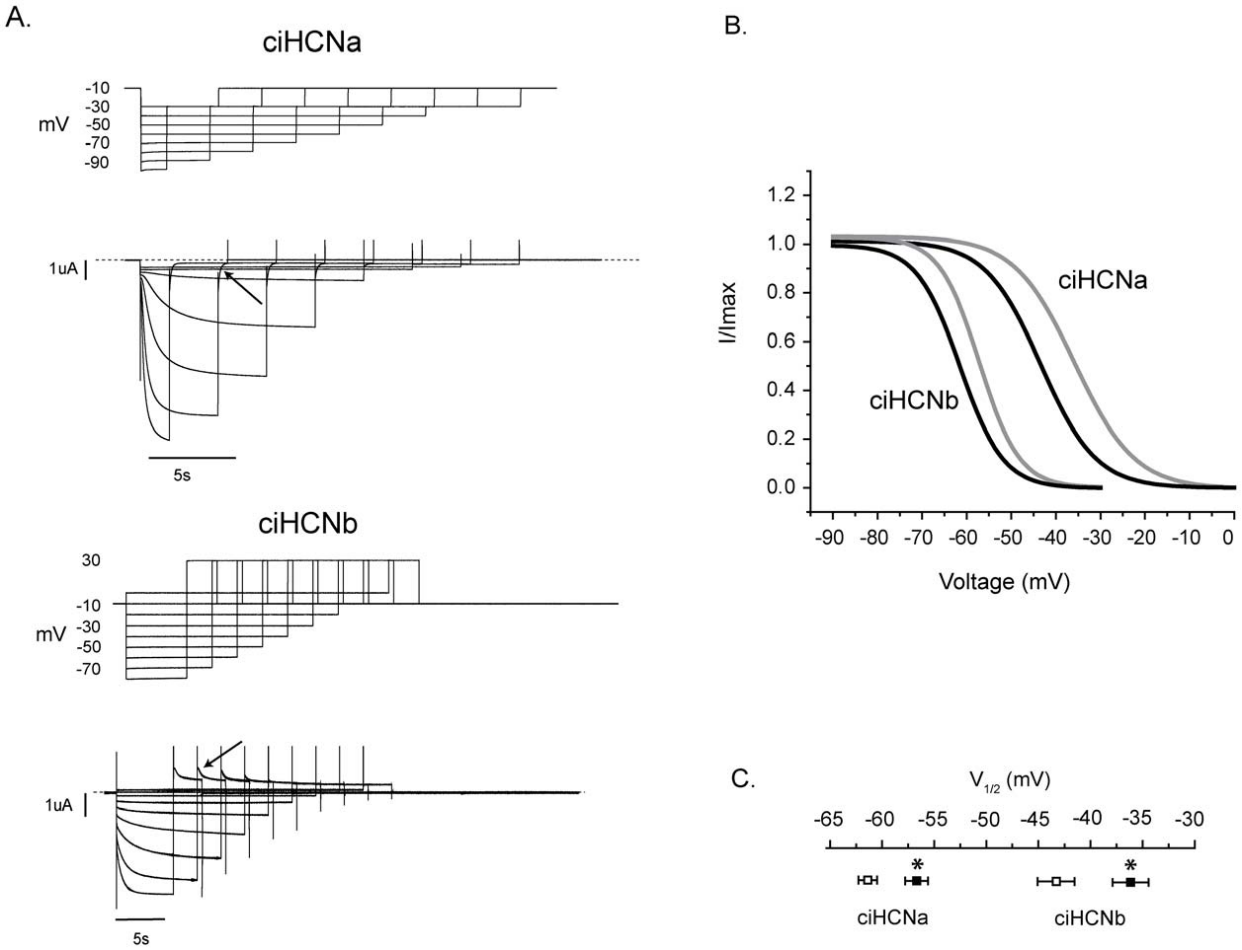
Opening of ciHCNs is facilitated by a rise in intracellular cAMP. A. Current traces for ciHCNa (above) and ciHCNb (below), elicited by the staggered voltage protocols shown above each set of current traces. “Tail” currents were elicited at −30 mV for ciHCNa and +30 mV for ciHCNb, and an example for each isoform is highlighted by a solid black arrow. B. Mean activation curves determined from single order Boltzmann fits of plots of normalized tail currents versus voltage from individual experiments and oocytes [35], under different conditions. The gray and black lines represent the mean curves obtained from control experiments and from experiments carried out in the presence of 10 mM 8-bromo cAMP, respectively. Number of experiments (representing one oocyte per experiment), without and with cAMP, were 14 and 11, respectively, for ciHCNa, and 8 and 8, respectively, for ciHCNb. C. Plot of mean V½ values ± s.e.m. determined by fitting the tail current data, obtained from individual experiments in oocytes expressing ciHCNa or ciHCNb in the absence (open squares) or presence of 8-Br-cAMP (filled squares), with a Boltzmann equation. *indicates significant difference (p<0.05, two-tailed unpaired t-test) between values obtained in control solution as compared to those obtained in a solution containing 8-Br-cAMP. The numbers of oocytes used per group are as for ‘B’.

To determine whether cAMP facilitates the opening of ciHCNs, the range over which I_h_ activates was established. Currents were elicited by a staggered set of hyperpolarizing test pulses, followed by tail currents in response to a second pulse invariant in voltage as shown in Figure 6A. The relative differences in the amplitudes of these tail currents are determined only by the degree of activation that occurs during the hyperpolarizing test pulses. Hyperpolarizing pulse lengths were longer at voltages at which the current required more time to approach a steady state. This staggered voltage protocol was performed on oocytes that had been incubated for 45 minutes in an extracellular solution containing 5 mM potassium either with or without 10 mM 8-bromo cAMP, a plasma membrane-permeable analog of cAMP. Current recordings were subsequently made in a 96 mM K^+^ solution to maximize current size. Tail currents, measured at −30 mV for ciHCNa and +30 mV for ciHCNb, were normalized, plotted versus test voltage and fitted using a Boltzmann equation (Figure 6B) from which values for mid-activation voltage (V½) and slope factor (k) were obtained.

To achieve a maximal level of intracellular cAMP, oocytes were incubated with 10 mM 8-Br-cAMP; this significantly shifted the activation curves by 7.1 mV and 7.4 mV for ciHCNa and ciHCNb, respectively (Figure 6C). These shifts in the activation curve are very close to the shift of 7.2 mV obtained by whole-cell patch clamp for HCN2 expressed in CHO cells using a maximal amount of cAMP (10 µM) in the patch pipette [45]. The average slope factor was analyzed from the same fitting procedure but, for both *Ciona* HCNs, it was not significantly affected by 8-bromo cAMP (data not shown).

Interestingly, the position of the activation curve, incubated in the control solution, was less negative in the oocytes expressing ciHCNb. The activation curve for both ciHCNs, in the absence of cAMP analog addition, is right shifted compared to those for HCN1 and HCN2 channels expressed in *Xenopus* oocytes [12].

### The time course of I_h_ activation is more complex for ciHCNa than for ciHCNb

**Figure 7:**
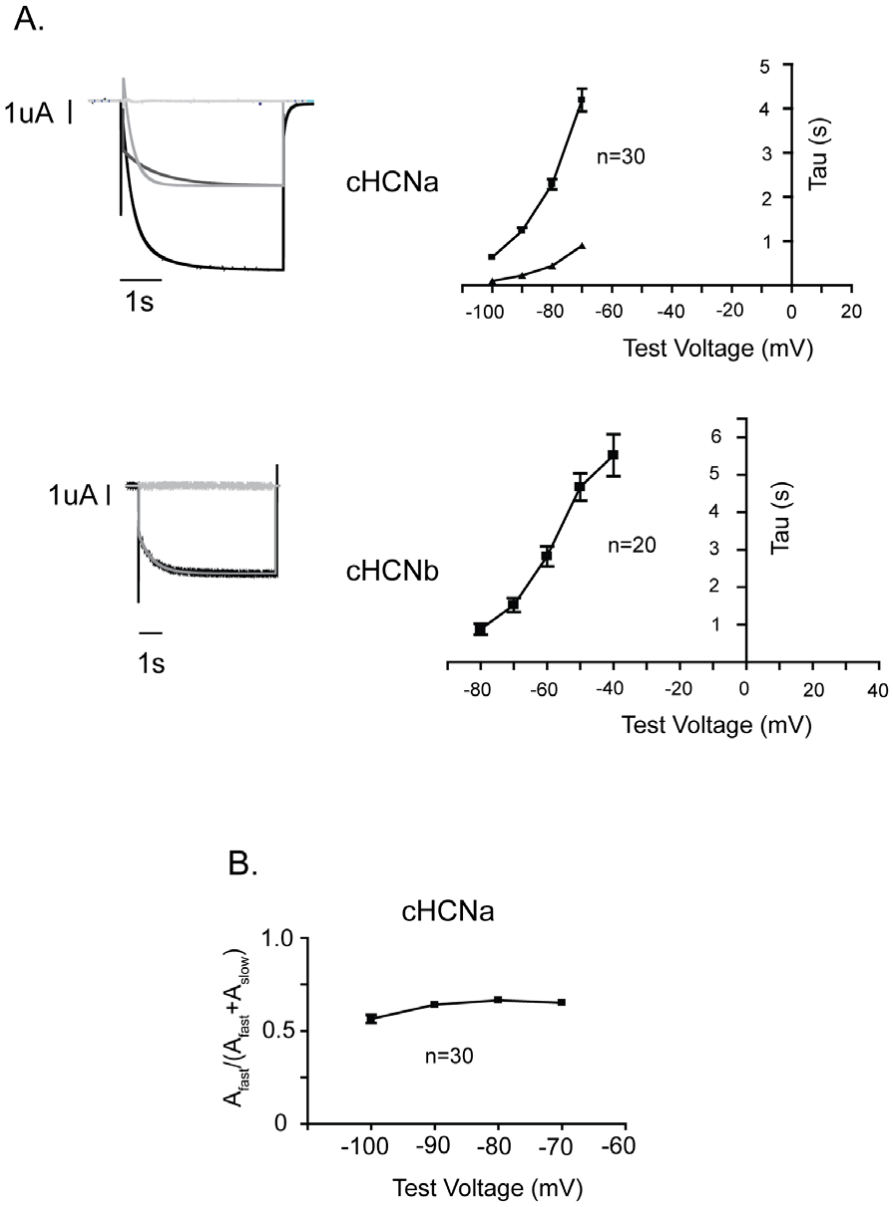
The time course of I_h_ activation and deactivation is different between ciHCNa and ciHCNb. A. (Left) Sample current traces elicited by a hyperpolarization of the membrane potential from a holding potential of −10 mV to −90 (ciHCNa) or −70 mV (ciHCNb). The slow portion of the current traces obtained by hyperpolarization to −90 or −70 mV (I_h_) was fit with a double (ciHCNa) or single (ciHCNb) exponential function. The sample fits of these traces are shown in dark gray, along with a plot of the residuals of the fits in light gray at the top of each current trace. (Right) Plots of Tau values, which were obtained from fitting with exponential functions, versus test voltage. B. Plot of the ratio of amplitudes of fast component versus the amplitudes of both the fast and slow component of the double exponential fit for the current traces, obtained from oocytes expressing ciHCNa as described in ‘A’, versus test voltage. For both ‘A’ and ‘B’, the values for “n” refer to the number of oocytes used. For all plots, the values represent the mean ± s.e.m.

Apparent from Figure 1 is the difference in the time course of I_h_ between ciHCNa and ciHCNb. To quantify this apparent difference, we determined the rates of I_h_ activation from the same staggered protocol used to ascertain the range of voltages over which opening occurred (Figure 7). For ciHCNa, I_h_ could be fit best using a double exponential function, with the slower component (τ) of fit reaching 4–5 seconds at its slowest. For ciHCNb, I_h_ was well-fitted with a single exponential function, with τ value reaching 5–6 seconds at its slowest.

Like ciHCNa, mammalian HCN1 and HCN2 channels activate with two components, but the contributions of each to the total current is different between these two channels. The amplitude of the fast component, when plotted as a proportion of the total amplitude, forms about 80% of the total across voltages ranging from approximately −110 to −60 mV for HCN1. In contrast, for HCN2, the proportion of the fast component forms approximately 80% of the total at −110 mV, but this decreases sharply to only 20% of the total at −75 mV. For ciHCNa, the amplitude of the fast component is relatively constant at ∼60% of the total current at voltages ranging from −100 mV to −70 mV (Figure 7C). Thus, based on the overall rates of activation for the two components and the insensitivity of the amplitude of the fast component as a proportion of the total current across voltage, the gating of the ciHCNa appears to be more similar to the HCN1 mammalian isoform than to any of the other three mammalian isoforms.

### I_inst_ and I_h_ flow through the ciHCNb channel pore in roughly equal amounts

An unusual feature of ciHCNb is the large size of I_inst_ that is apparent at all voltages examined. This large size may be due to inadequate closing of the channel gate or because the holding potential used to examine I_inst_ and I_h_ (−10 mV) is still within the range of channel opening by voltage i.e. the channel is still somewhat open at that voltage. This can be ruled out by an examination of Figure 7A. This figure shows that, for ciHCNb, a large amount of outward current remains after deactivation of outward tail currents, which were elicited at +30 mV. Thus, the data favors the former explanation, namely that the gate fails to close across a wide range of voltages.

**Figure 8:**
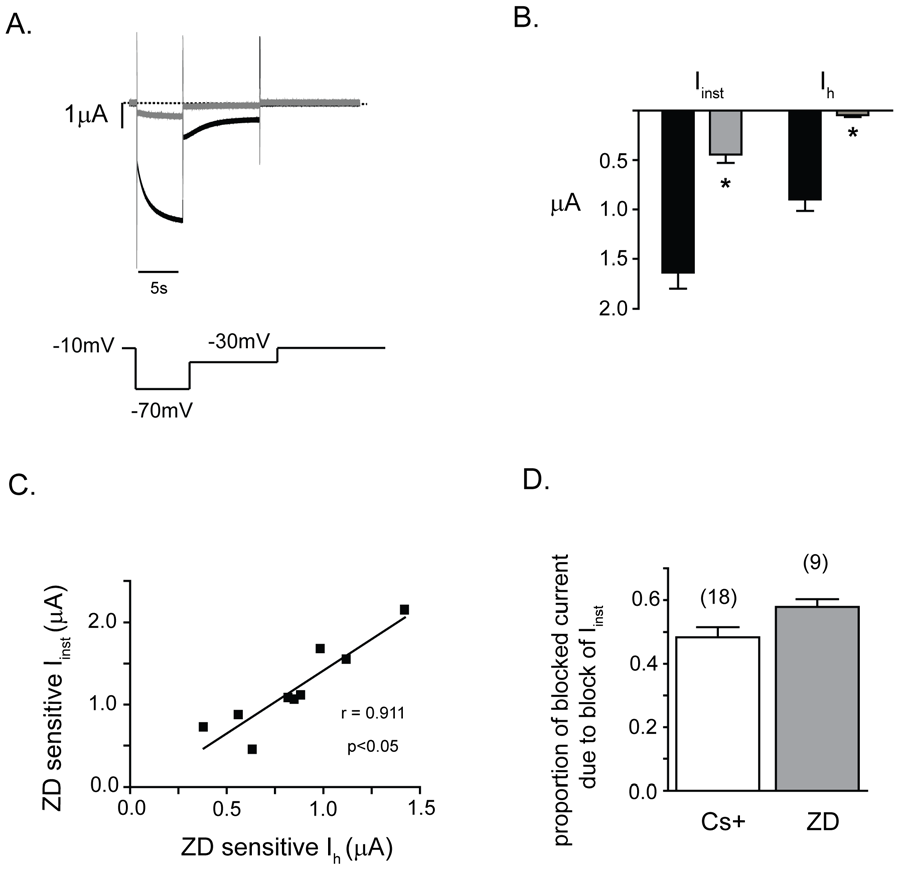
I_inst_ and I_h_ flow through the ciHCNb pore in approximately the same proportion. A. Current traces elicited by a hyperpolarization of the membrane potential from a holding potential of −10 mV to −70 mV before (black trace) or after (gray trace) a 15 min incubation with ZD7288. The complete voltage protocol is shown below the current traces. The dotted line represents the zero current level. B. Bar graph of I_inst_ and I_h_ before (black bars) and after (gray bars) 15 minute incubation with ZD7288, as carried out in ‘A’. * indicates a significant block of the current component by this agent (p<0.05 in a two-tailed paired t-test). C. Plot of ZD7288-sensitive I_inst_ versus I_h_. Black line represents a linear correlation, which was a significant (r = 0.9, p<0.05). D. Bar graph showing the ratio of I_inst_ over total current (I_inst_+I_h_) that is blocked by Cs+ or ZD7288; this ratio represents the proportion of blocked current that is due to block of I_inst_. An example of the Cs+ data used for this figure is shown in Figure 2. In ‘B’ and ‘D’, values represent mean ± s.e.m and the numbers in brackets are the numbers of oocytes used. The ZD7288 data in ‘B’, ‘C’ and ‘D’ come from the same 9 oocytes.

To further confirm that I_inst_ flows through ciHCNb, we blocked it with ZD7288 (Figure 8), a highly specific pore- blocker of HCN channels [46], [47], [48], [49], [50]. Indeed, ZD788 effectively blocked both I_inst_ and I_h_ in oocytes expressing ciHCNb (Figure 8 A,B). A plot of the amplitudes of ZD-sensitive I_h_ versus I_inst_ from the same voltage pulses showed a high correlation between them (Figure 8C). Together, the block of I_inst_ and I_h_ by cesium (Fig. 2) and ZD7288, and the high degree of correlation in amplitudes between the respective ZD-sensitive components, are strong evidence that both currents result from ions flowing specifically through the ciHCNb channel pore. Finally, when we found that, at −70 mV, I_inst_ formed about half of the total current that was sensitive to either Cs^+^ or ZD7288 (Figure 8D).

## Discussion

To help establish the pattern of HCN evolution from invertebrates to vertebrates, we have refined the phylogeny of HCNs, and cloned and functionally characterized two HCN channels from *Ciona intestinalis*. ciHCNa undergoes N-glycosylation at a sequon near the pore and functions similarly to the mammalian HCNs. In contrast, ciHCNb does not undergo N-glycosylation and, unlike both ciHCNa and ciHCNc, it does not possess the pore-associated sequon. Together with the presence of that sequon in all known vertebrates and *Oikoplerua dioica* and its absence from all known invertebrates [19], [20], our findings to date suggest that the sequon was present an ancestor common to both vertebrates and tunicates and lost in ciHCNb. CiHCNb also possesses an unusual gating phenotype in which the channel fails to close completely. Other key features of HCN function, including hyperpolarization-sensitive opening, cAMP facilitation of opening, permeability to sodium and potassium and block of ion flow by cesium are retained in both *Ciona* HCNs.

An instantaneous, or voltage-independent, conductance through HCNs has been appreciated only relatively recently, since they were cloned and expressed heterologously in *Xenopus* oocytes and mammalian cell lines, where it could be separated from similar endogenous currents and reliably studied [4], [9], [35], [36], [47], [51], [52]. In most known HCNs, I_inst_ represents only a very small fraction of the total ion flow. A somewhat larger I_inst_ has been observed in SpIH, one of two HCNs found in sea urchin, that represents about four percent of the total current flowing through it [47]. In contrast, I_inst_ makes up about half of the total current through ciHCNb channel, a unique adaptation that likely has dramatic consequences *in vivo*; in cells expressing ciHCNb, a constant depolarizing influence in the absence of any hyperpolarizing stimulus might be predicted. Structural insight into the origins of I_inst_ opening has come from studies using a combined electrophysiological and mutagenesis approach, which have shown that the region connecting the putative fourth and fifth transmembrane segments of HCNs controls the degree of voltage-insensitive opening [36], [52]; this region is highly divergent between ciHCNa and ciHCNb and will be a focus of future investigations of the structural basis for this gating phenotype. Interestingly, the HCN-related hyperpolarization-activated potassium channels from plants (e.g. KAT1, AKT1 and AKT2) also demonstrate variable proportions of slow and instantaneous currents among them [53], [54], [55], suggesting that this mode of gating was also integral in a common ancestral ion channel.

Tunicates diverged from a common ancestor with vertebrates over 550MYA and have undergone rapid evolution that includes dramatic rearrangement and loss of genomic material [24], [56], [57], [58], [59]. Selective and rapid evolution could explain the low level of sequence similarity between the two *Ciona* HCNs compared to that among the mammalian HCNs, the fact that they are as similar to each as they are to the vertebrate HCNs, the pronounced differences in function found between them and the absence of the N-glycosylation sequon in ciHCNb; this could also explain the differences in exon boundary structure between them, which is again unlike the vertebrate HCN paralogs that completely share these boundaries [19]. Finally, functions other than those that can be measured in an artificial expression system, e.g. distribution of the isoforms in specific cell types and their interactions with specific molecular partners, may also differ between the two *Ciona* HCNs. The significance of the differences in coding and regulatory regions of the *Ciona* HCN genes remains to be studied. An examination of the channels *in vivo* will help to define the respective roles for the *Ciona* HCN isoforms. A putative ortholog of ciHCNa was recently identified in a related tunicate species, *Botryllus schlosseri* [60] and was shown to be strongly expressed and functional in the cardiac pacemaking tissue. When divergence of both lines is considered, over one billion years of independent evolution have occurred between the extant ascidians and modern vertebrates. Features shared between the tunicate and mammalian channels can shed light on the ancestral condition [57] of HCN channel function and the role for these genes prior to the family expansion in the vertebrate lineage.

## References

[1] Robinson RB, Siegelbaum SA (2003) Hyperpolarization-activated cation currents: from molecules to physiological function. Annu Rev Physiol 65: 453–480. 12471170 10.1146/annurev.physiol.65.092101.142734

[2] Ludwig A, Zong X, Jeglitsch M, Hofmann F, Biel M (1998) A family of hyperpolarization-activated mammalian cation channels. Nature 393: 587–591. 9634236 10.1038/31255

[3] Santoro B, Liu DT, Yao H, Bartsch D, Kandel ER, et al (1998) Identification of a gene encoding a hyperpolarization-activated pacemaker channel of brain. Cell 93: 717–729. 9630217 10.1016/s0092-8674(00)81434-8

[4] Ishii TM, Takano M, Xie LH, Noma A, Ohmori H (1999) Molecular characterization of the hyperpolarization-activated cation channel in rabbit heart sinoatrial node. J Biol Chem 274: 12835–12839. 10212270 10.1074/jbc.274.18.12835

[5] Marx T, Gisselmann G, Stortkuhl KF, Hovemann BT, Hatt H (1999) Molecular cloning of a putative voltage- and cyclic nucleotide-gated ion channel present in the antennae and eyes of Drosophila melanogaster. Invert Neurosci 4: 55–63. 12491074 10.1007/pl00022368

[6] Gisselmann G, Warnstedt M, Gamerschlag B, Bormann A, Marx T, et al (2003) Characterization of recombinant and native Ih-channels from Apis mellifera. Insect Biochem Mol Biol 33: 1123–1134. 14563363 10.1016/s0965-1748(03)00132-2

[7] Gisselmann G, Marx T, Bobkov Y, Wetzel CH, Neuhaus EM, et al (2005) Molecular and functional characterization of an I(h)-channel from lobster olfactory receptor neurons. Eur J Neurosci 21: 1635–1647. 15845091 10.1111/j.1460-9568.2005.03992.x

[8] Ouyang Q, Goeritz M, Harris-Warrick RM (2007) Panulirus interruptus Ih-channel gene PIIH: modification of channel properties by alternative splicing and role in rhythmic activity. J Neurophysiol 97: 3880–3892. 17409170 10.1152/jn.00246.2007

[9] Gauss R, Seifert R, Kaupp UB (1998) Molecular identification of a hyperpolarization-activated channel in sea urchin sperm. Nature 393: 583–587. 9634235 10.1038/31248

[10] Galindo BE, Neill AT, Vacquier VD (2005) A new hyperpolarization-activated, cyclic nucleotide-gated channel from sea urchin sperm flagella. Biochem Biophys Res Commun 334: 96–101. 15992765 10.1016/j.bbrc.2005.06.074

[11] Baruscotti M, Bucchi A, Difrancesco D (2005) Physiology and pharmacology of the cardiac pacemaker (“funny”) current. Pharmacol Ther 107: 59–79. 15963351 10.1016/j.pharmthera.2005.01.005

[12] Santoro B, Chen S, Luthi A, Pavlidis P, Shumyatsky GP, et al (2000) Molecular and functional heterogeneity of hyperpolarization-activated pacemaker channels in the mouse CNS. J Neurosci 20: 5264–5275. 10884310 10.1523/JNEUROSCI.20-14-05264.2000 PMC6772310

[13] Altomare C, Bucchi A, Camatini E, Baruscotti M, Viscomi C, et al (2001) Integrated allosteric model of voltage gating of HCN channels. J Gen Physiol 117: 519–532. 11382803 10.1085/jgp.117.6.519 PMC2232403

[14] Chen S, Wang J, Siegelbaum SA (2001) Properties of hyperpolarization-activated pacemaker current defined by coassembly of HCN1 and HCN2 subunits and basal modulation by cyclic nucleotide. J Gen Physiol 117: 491–504. 11331358 10.1085/jgp.117.5.491 PMC2233656

[15] Moosmang S, Stieber J, Zong X, Biel M, Hofmann F, et al (2001) Cellular expression and functional characterization of four hyperpolarization-activated pacemaker channels in cardiac and neuronal tissues. Eur J Biochem 268: 1646–1652. 11248683 10.1046/j.1432-1327.2001.02036.x

[16] Mistrik P, Mader R, Michalakis S, Weidinger M, Pfeifer A, et al (2005) The murine HCN3 gene encodes a hyperpolarization-activated cation channel with slow kinetics and unique response to cyclic nucleotides. J Biol Chem 280: 27056–27061. 15923185 10.1074/jbc.M502696200

[17] Stieber J, Stockl G, Herrmann S, Hassfurth B, Hofmann F (2005) Functional expression of the human HCN3 channel. J Biol Chem 280: 34635–34643. 16043489 10.1074/jbc.M502508200

[18] Shin KS, Maertens C, Proenza C, Rothberg BS, Yellen G (2004) Inactivation in HCN channels results from reclosure of the activation gate: desensitization to voltage. Neuron 41: 737–744. 15003173 10.1016/s0896-6273(04)00083-2

[19] Jackson HA, Marshall CR, Accili EA (2007) Evolution and structural diversification of hyperpolarization-activated cyclic nucleotide-gated channel genes. Physiol Genomics 29: 231–245. 17227887 10.1152/physiolgenomics.00142.2006

[20] Hegle AP, Nazzari H, Roth A, Angoli D, Accili EA (2010) Evolutionary emergence of N-glycosylation as a variable promoter of HCN channel surface expression. Am J Physiol Cell Physiol 298: C1066–1076. 20130205 10.1152/ajpcell.00389.2009

[21] Much B, Wahl-Schott C, Zong X, Schneider A, Baumann L, et al (2003) Role of subunit heteromerization and N-linked glycosylation in the formation of functional hyperpolarization-activated cyclic nucleotide-gated channels. J Biol Chem 278: 43781–43786. 12928435 10.1074/jbc.M306958200

[22] Suzuki MM, Nishikawa T, Bird A (2005) Genomic approaches reveal unexpected genetic divergence within Ciona intestinalis. J Mol Evol 61: 627–635. 16205978 10.1007/s00239-005-0009-3

[23] Satoh N, Satou Y, Davidson B, Levine M (2003) Ciona intestinalis: an emerging model for whole-genome analyses. Trends Genet 19: 376–381. 12850442 10.1016/S0168-9525(03)00144-6

[24] Delsuc F, Brinkmann H, Chourrout D, Philippe H (2006) Tunicates and not cephalochordates are the closest living relatives of vertebrates. Nature 439: 965–968. 16495997 10.1038/nature04336

[25] Corbo JC, Di Gregorio A, Levine M (2001) The ascidian as a model organism in developmental and evolutionary biology. Cell 106: 535–538. 11551501 10.1016/s0092-8674(01)00481-0

[26] Okamura Y, Nishino A, Murata Y, Nakajo K, Iwasaki H, et al (2005) Comprehensive analysis of the ascidian genome reveals novel insights into the molecular evolution of ion channel genes. Physiol Genomics 22: 269–282. 15914577 10.1152/physiolgenomics.00229.2004

[27] Karolchik D, Kuhn RM, Baertsch R, Barber GP, Clawson H, et al (2008) The UCSC Genome Browser Database: 2008 update. Nucleic Acids Res 36: D773–779. 18086701 10.1093/nar/gkm966 PMC2238835

[28] Thompson JD, Gibson TJ, Plewniak F, Jeanmougin F, Higgins DG (1997) The CLUSTAL_X windows interface: flexible strategies for multiple sequence alignment aided by quality analysis tools. Nucleic Acids Res 25: 4876–4882. 9396791 10.1093/nar/25.24.4876 PMC147148

[29] Tamura K, Peterson D, Peterson N, Stecher G, Nei M, et al (2011) MEGA5: molecular evolutionary genetics analysis using maximum likelihood, evolutionary distance, and maximum parsimony methods. Mol Biol Evol 28: 2731–2739. 21546353 10.1093/molbev/msr121 PMC3203626

[30] Saitou N, Nei M (1987) The neighbor-joining method: a new method for reconstructing phylogenetic trees. Mol Biol Evol 4: 406–425. 3447015 10.1093/oxfordjournals.molbev.a040454

[31] Dehal P, Satou Y, Campbell RK, Chapman J, Degnan B, et al (2002) The draft genome of Ciona intestinalis: insights into chordate and vertebrate origins. Science 298: 2157–2167. 12481130 10.1126/science.1080049

[32] Jegla T, Salkoff L (1997) A novel subunit for shal K+ channels radically alters activation and inactivation. J Neurosci 17: 32–44. 8987734 10.1523/JNEUROSCI.17-01-00032.1997 PMC6793676

[33] Zhang X, Bursulaya B, Lee CC, Chen B, Pivaroff K, et al (2009) Divalent cations slow activation of EAG family K+ channels through direct binding to S4. Biophys J 97: 110–120. 19580749 10.1016/j.bpj.2009.04.032 PMC2711382

[34] Blair JE, Hedges SB (2005) Molecular phylogeny and divergence times of deuterostome animals. Mol Biol Evol 22: 2275–2284. 16049193 10.1093/molbev/msi225

[35] Proenza C, Angoli D, Agranovich E, Macri V, Accili EA (2002) Pacemaker channels produce an instantaneous current. J Biol Chem 277: 5101–5109. 11741901 10.1074/jbc.M106974200

[36] Macri V, Accili EA (2004) Structural elements of instantaneous and slow gating in hyperpolarization-activated cyclic nucleotide-gated channels. J Biol Chem 279: 16832–16846. 14752094 10.1074/jbc.M400518200

[37] DiFrancesco D (1982) Block and activation of the pace-maker channel in calf purkinje fibres: effects of potassium, caesium and rubidium. J Physiol 329: 485–507. 6292407 10.1113/jphysiol.1982.sp014315 PMC1224792

[38] Macri V, Proenza C, Agranovich E, Angoli D, Accili EA (2002) Separable gating mechanisms in a Mammalian pacemaker channel. J Biol Chem 277: 35939–35946. 12121985 10.1074/jbc.M203485200

[39] DiFrancesco D (1981) A study of the ionic nature of the pace-maker current in calf Purkinje fibres. J Physiol 314: 377–393. 6273534 10.1113/jphysiol.1981.sp013714 PMC1249440

[40] DiFrancesco D (1981) A new interpretation of the pace-maker current in calf Purkinje fibres. J Physiol 314: 359–376. 6273533 10.1113/jphysiol.1981.sp013713 PMC1249439

[41] Zagotta WN, Olivier NB, Black KD, Young EC, Olson R, et al (2003) Structural basis for modulation and agonist specificity of HCN pacemaker channels. Nature 425: 200–205. 12968185 10.1038/nature01922

[42] DiFrancesco D, Tortora P (1991) Direct activation of cardiac pacemaker channels by intracellular cyclic AMP. Nature 351: 145–147. 1709448 10.1038/351145a0

[43] Wainger BJ, DeGennaro M, Santoro B, Siegelbaum SA, Tibbs GR (2001) Molecular mechanism of cAMP modulation of HCN pacemaker channels. Nature 411: 805–810. 11459060 10.1038/35081088

[44] Viscomi C, Altomare C, Bucchi A, Camatini E, Baruscotti M, et al (2001) C terminus-mediated control of voltage and cAMP gating of hyperpolarization-activated cyclic nucleotide-gated channels. J Biol Chem 276: 29930–29934. 11397812 10.1074/jbc.M103971200

[45] DiFrancesco JC, Barbuti A, Milanesi R, Coco S, Bucchi A, et al Recessive loss-of-function mutation in the pacemaker HCN2 channel causing increased neuronal excitability in a patient with idiopathic generalized epilepsy. J Neurosci 31: 17327–17337. 10.1523/JNEUROSCI.3727-11.2011 PMC6623833 22131395

[46] BoSmith RE, Briggs I, Sturgess NC (1993) Inhibitory actions of ZENECA ZD7288 on whole-cell hyperpolarization activated inward current (If) in guinea-pig dissociated sinoatrial node cells. Br J Pharmacol 110: 343–349. 7693281 10.1111/j.1476-5381.1993.tb13815.x PMC2176028

[47] Proenza C, Yellen G (2006) Distinct populations of HCN pacemaker channels produce voltage-dependent and voltage-independent currents. J Gen Physiol 127: 183–190. 16446506 10.1085/jgp.200509389 PMC2151495

[48] Shin KS, Rothberg BS, Yellen G (2001) Blocker state dependence and trapping in hyperpolarization-activated cation channels: evidence for an intracellular activation gate. J Gen Physiol 117: 91–101. 11158163 10.1085/jgp.117.2.91 PMC2217248

[49] Cheng L, Kinard K, Rajamani R, Sanguinetti MC (2007) Molecular mapping of the binding site for a blocker of hyperpolarization-activated, cyclic nucleotide-modulated pacemaker channels. J Pharmacol Exp Ther 322: 931–939. 17578902 10.1124/jpet.107.121467

[50] Qu Y, Whitaker GM, Hove-Madsen L, Tibbits GF, Accili EA (2008) Hyperpolarization-activated cyclic nucleotide-modulated ‘HCN’ channels confer regular and faster rhythmicity to beating mouse embryonic stem cells. J Physiol 586: 701–716. 18033814 10.1113/jphysiol.2007.144329 PMC2375615

[51] Chen J, Mitcheson JS, Lin M, Sanguinetti MC (2000) Functional roles of charged residues in the putative voltage sensor of the HCN2 pacemaker channel. J Biol Chem 275: 36465–36471. 10962006 10.1074/jbc.M007034200

[52] Chen J, Mitcheson JS, Tristani-Firouzi M, Lin M, Sanguinetti MC (2001) The S4–S5 linker couples voltage sensing and activation of pacemaker channels. Proc Natl Acad Sci U S A 98: 11277–11282. 11553787 10.1073/pnas.201250598 PMC58720

[53] Schachtman DP, Schroeder JI, Lucas WJ, Anderson JA, Gaber RF (1992) Expression of an inward-rectifying potassium channel by the Arabidopsis KAT1 cDNA. Science 258: 1654–1658. 8966547 10.1126/science.8966547

[54] Gaymard F, Cerutti M, Horeau C, Lemaillet G, Urbach S, et al (1996) The baculovirus/insect cell system as an alternative to Xenopus oocytes. First characterization of the AKT1 K+ channel from Arabidopsis thaliana. J Biol Chem 271: 22863–22870. 8798465 10.1074/jbc.271.37.22863

[55] Lacombe B, Pilot G, Michard E, Gaymard F, Sentenac H, et al (2000) A shaker-like K(+) channel with weak rectification is expressed in both source and sink phloem tissues of Arabidopsis. Plant Cell 12: 837–851. 10852932 10.1105/tpc.12.6.837 PMC149088

[56] Swalla BJ, Cameron CB, Corley LS, Garey JR (2000) Urochordates are monophyletic within the deuterostomes. Syst Biol 49: 52–64. 12116483 10.1080/10635150050207384

[57] Gee H (2006) Evolution: careful with that amphioxus. Nature 439: 923–924. 16495981 10.1038/439923a

[58] Schubert M, Escriva H, Xavier-Neto J, Laudet V (2006) Amphioxus and tunicates as evolutionary model systems. Trends Ecol Evol 21: 269–277. 16697913 10.1016/j.tree.2006.01.009

[59] Putnam NH, Butts T, Ferrier DE, Furlong RF, Hellsten U, et al (2008) The amphioxus genome and the evolution of the chordate karyotype. Nature 453: 1064–1071. 18563158 10.1038/nature06967

[60] Hellbach A, Tiozzo S, Ohn J, Liebling M, De Tomaso AW (2011) Characterization of HCN and cardiac function in a colonial ascidian. J Exp Zool A Ecol Genet Physiol 315: 476–486. 21770038 10.1002/jez.695

